# Prostaglandin D2 synthase is differentially methylated specifically in Huntington’s Disease

**DOI:** 10.1002/alz.084097

**Published:** 2025-01-03

**Authors:** Greg Wheildon, Luke Weymouth, Adam Smith, Rebecca G. Smith, Claire Troakes, Safa Al‐Sarraj, Katie Lunnon

**Affiliations:** ^1^ University of Exeter, Exeter United Kingdom; ^2^ King’s College London, MRC London Neurodegenerative Diseases Brain Bank, London United Kingdom; ^3^ Kings College NHS Foundation Trust, London United Kingdom; ^4^ University of Exeter, Exeter, Devon United Kingdom

## Abstract

**Background:**

Huntington’s disease (HD) is an autosomal dominant condition causing severe neurodegeneration in the striatum and the entorhinal cortex (EC). An epigenome wide association study of DNA methylation in HD by our group, identified potential hypomethylation at the *PTGDS* gene in the striatum. We aimed to validate this result through pyrosequencing, examining the locus in fine detail, and to assess the signal specificity by profiling multiple neurodegenerative diseases.

**Method:**

379 EC and striatal DNA samples from 85 control, 73 Alzheimer’s disease (AD), 90 dementia with Lewy bodies (DLB), 20 HD, 34 Parkinson’s disease (PD) and 24 vascular dementia (VaD) subjects were matched for sex and age. DNA was bisulfite converted, before *PTGDS* target region amplification and subsequent pyrosequencing. Samples were analysed using linear models comparing the control group to each disease, controlling for sex, age, and batch effects. Average methylation levels across the amplicon were also compared. Additional analysis stratifying DLB and VaD cases by co‐existing AD pathology was also performed.

**Result:**

We confirmed our previous findings of DNA hypomethylation at *PTGDS* in the striatum in HD. Furthermore, we found significant hypomethylation in the targeted region, both at the level of individual CpG sites, and across the amplicon in the EC. We also observed nominally significant changes in PD and DLB with co‐existing AD pathology across the amplicon, but these did not pass multiple correction.

**Conclusion:**

This study validates our previous findings of *PTGDS* hypomethylation in HD in the striatum and presents novel associations at the EC. Together, these findings suggest further investigation of *PTGDS*, given others report differential expression in HD oligodendrocytes. Future studies should address the cell‐specific profile of *PTGDS* methylation and its transcriptional regulation in HD through fluorescent activated cell sorting.

